# Weight Gain and Nutrition during Pregnancy: An Analysis of Clinical Practice Guidelines in the Asia-Pacific Region

**DOI:** 10.3390/nu14061288

**Published:** 2022-03-18

**Authors:** Tomoko Aoyama, Donglai Li, Jacquie Lindsay Bay

**Affiliations:** 1 Liggins Institute, University of Auckland, Auckland 1142, New Zealand; j.bay@auckland.ac.nz; 2 National Institutes of Biomedical Innovation, Health and Nutrition, Tokyo 162-8636, Japan; 3 Faculty of Science, University of Auckland, Auckland 1142, New Zealand; dli076@aucklanduni.ac.nz

**Keywords:** gestational weight gain, maternal nutrition, pregnancy, clinical practice guideline

## Abstract

Nutrition and weight gain during pregnancy can influence the life-course health of offspring. Clinical practice guidelines play an important role in ensuring appropriate nutrition and weight gain among pregnant women. This study aims to identify clinical practice guidelines on gestational weight gain and/or maternal nutrition across the Asia-Pacific region and to determine the quality of the guidelines and variability in the recommendations. Through a systematic search of grey literature from 38 Asia-Pacific countries, 23 published guidelines were obtained. Of these, 10 eligible clinical practice guidelines reporting nutrition- or/and weight-related recommendations for pregnant women were selected and reviewed. Guideline quality was determined using the Assessment of Guidelines for Research Evaluation II (AGREE II) instrument. Of the 10 guidelines, 90% were classified as low-quality in the AGREE II appraisal. Several variations were found with respect to recommendations on gestational weight gain, including those specific to Asian populations. The recommendations on dietary advice, additional energy intake, and nutritional supplementation during pregnancy were varied. Clinical practice guidelines on weight gain and nutrition in pregnancy across the Asia-Pacific region are generally of poor quality, reflecting significant variation, and need to be improved to ensure pregnant women receive appropriate advice. (PROSPERO registration no. CRD42021291395).

## 1. Introduction

Pregnancy is one of the most important periods that can influence the lifelong health of the offspring [1]. Accumulating evidence from animal studies has demonstrated that various maternal nutritional exposures, including both under- and over-nutrition during pregnancy, have a direct effect on size and body composition at birth and cause long-lasting effects on metabolic responses in adulthood [2]. In humans, a number of epidemiological studies support the fact that nutritional status during pregnancy is associated with foetal development, which in turn is associated with the risk of the child developing cardiovascular and metabolic disorders in later life. For instance, inadequate gestational weight gain (GWG) is associated with increased risk of low birthweight infants and small-for-gestational-age infants [3,4], which are themselves associated with an increased risk of cardiovascular and metabolic disorders in their future life [5]. On the other hand, excessive GWG increases the risk of macrosomia and a large-for-gestational-age [4,6], which likely are associated with obesity and related chronic diseases in adulthood [7]. Pregnancy is therefore a critical period that may amplify the intergenerational spread of obesity and non-communicable diseases. As this intergenerational impact of nutrition is gradually being recognised, the importance of addressing aspects of early nutrition to reduce health inequalities in the next generation through maternal and child nutrition has become a major concern [1].

The associations between inappropriate GWG and adverse outcomes in infants have been reported worldwide and across ethnicities [4]. Weight gain and nutrition during pregnancy is therefore a key public health issue, indicating the need for guidelines at national and international levels that include recommendations for optimising GWG and maternal nutrition. Although many forms of guidelines have been published, clinical practice guidelines (CPGs) play an important role in implementing scientific evidence into clinical practice. CPGs are defined as ‘systematically developed statements about specific clinical problems intended to assist practitioners and patients in making decisions about appropriate health care’ [8]. Therefore, the quality of CPGs for GWG and maternal nutrition is important in determining the health of pregnant women and their future children in a given country.

To date, three studies have attempted to systematically search for and review guidelines on GWG and/or maternal nutrition [9,10,11], including two studies that analysed a wide range of guidelines that do not fall fully into the CPG category [9,10]. Only one study searched for and evaluated CPGs [11], but mostly included CPGs from high- and middle-income countries, such as Europe and the United States, with a significant lack of CPGs from low- and middle-income countries. Only CPGs from Australia and New Zealand in the Asia-Pacific region were included [11]. However, as CPGs can fall under the category of ‘grey literature’ [12], which is not identified through an ordinal systematic literature search [13], this may not mean that CPGs do not exist. Different approaches to searching and accessing grey (unpublished) literature sources are needed to better understand the availability of CPGs in this region.

In addition, little is still known about the quality of CPGs and the variability of recommendations on GWG and maternal nutrition across the Asia-Pacific region. Therefore, the aim of this study is to assess the quality of CPGs and the variation in recommendations and to provide an overview of the current state of CPGs for GWG and maternal nutrition in the Asia-Pacific region.

## 2. Materials and Methods

The systematic review protocol was developed and registered with PROSPERO (CRD42021291395). The PICAR (population and clinical area(s), intervention(s), comparator(s), comparison(s), and (key) content, attributes of CPGs, and recommendation characteristics) framework [14] was developed based on the previous study [11] and is shown in Table 1. The present study covered 11 South-East Asian and 27 Western Pacific countries, which are members of the World Health Organization (WHO) [15] as of December 2020. Given that such countries’ guidelines are mainly ‘grey literature’ that are not identified by the systematic bibliography search, other search strategies were developed to identify national and international guidelines in the Asia-Pacific region, including browsing government websites, inquiring with government agencies, and performing hand searches [13].

First, in December 2020, we accessed each national government website, including the ‘Ministry of Health’ with a limit on the domain, and sought to identify web pages presenting guidelines on GWG and/or maternal nutrition for each listed Asia-Pacific country. Government agencies were contacted directly via e-mail or websites in January 2021 if such guidelines were not identified by searching the websites. If any references on GWG and/or maternal nutrition were not acquired by the above processes, we conducted a manual search for guidelines through search engines (e.g., Bing, Google) in late January 2021. During this manual search process, some language support was obtained from native speakers with academic backgrounds to identify non-English resources, for which English searches yielded little information (China, Korea, Japan, and Indonesia). Guidelines identified by previous studies [9,10,11] were also accessed. For resources written in Chinese, Indonesian, and Vietnamese, titles and abstracts were translated using Google Translate for screening, and the full texts were translated to English by a translation company located in Japan to enable eligibility checks. The resources written in Japanese were translated into English by an author (T.A.).

Two authors (D.L. and T.A.) independently screened the titles and abstracts (if available) and then screened the full text of the records independently for eligibility. We used the inclusion and exclusion criteria used by Grammatikopoulou et al. [11], with minor modifications. We included: (1) National and international CPGs, including consensus papers or practice papers; (2) in a full-text format that is publicly available; (3) published since the year 2000 in any language; (4) issued from professional or governmental organisations in the Asia-Pacific region; (5) reporting nutrition- and/or GWG-related recommendations; (6) intended for health professionals; (7) latest version; and (8) with no restrictions on their quality. The following were excluded: (1) Guidelines on diet/nutrition for the general public (e.g., dietary guidelines or dietary reference intakes); (2) guidelines focusing on the intake of single nutrients or substances; (3) guidelines on the care of pregnant women with obesity, gestational diabetes, pregnancy-induced hypertension, or any other concomitant chronic disease, and (4) guidelines intended for patients and other end users but not for healthcare professionals, as these lacked rigour of development or information on stakeholder involvement. Any disagreements between individual judgements were resolved through discussion. The reasons for inclusion and exclusion were shared using a spreadsheet between the authors, and a consensus was reached. Documents meeting all the inclusion criteria without meeting any exclusion criteria were selected for critical appraisal using the Appraisal of Guidelines for Research and Evaluation Instrument (AGREE II), a validated tool to assess the methodological rigour and transparency of CPGs [11,16].

As part of the review process, one author (T.A.) extracted the data into an Excel spreadsheet, and another (D.L.) independently verified the results for accuracy and completeness. To synthesise information on recommendations for weight gain and nutrition during pregnancy, data on the following items were extracted: (1) Characteristics of the CPGs: country, year of publication, title of guideline, and language; (2) GWG recommendations and evidence base behind each recommendation; and (3) nutritional recommendations, including dietary advice (foods to choose and to avoid or limit), additional energy intake (kcal/day), and nutritional supplementation during pregnancy.

Two authors (T.A. and D.L.) appraised the CPGs independently using AGREE II. The six domains (scope and purpose, stakeholder involvement, rigour of development, clarity of presentation, applicability, and editorial independence), including 23 individual items for guideline quality, were assessed, as well as the overall assessments score and suggestions for using the guideline (‘recommend without modifications’, ‘recommend with modifications’, or ‘not recommend’). Each of the 23 items was assigned a score from 1 (strongly disagree) to 7 (strongly agree) based on AGREE II. Then six domain scores were calculated by summing up all the scores of the individual items in a domain and by scaling the total as a percentage (0–100%) of the maximum possible score for that domain. As the AGREE II does not include explicit cut-off levels to determine levels of overall guideline quality, a 3-step cut-off system was applied based on previous studies [17,18,19]. Guidelines were classified based on their domain scores, with all domains being given equal importance: high quality if all domains scored ≥70%; moderate quality if any domain scored ≥50% and <70%, and low quality if any domain scored <50% [19].

## 3. Results

### 3.1. Characteristics and Quality of Selected CPGs

**Figure 1:**
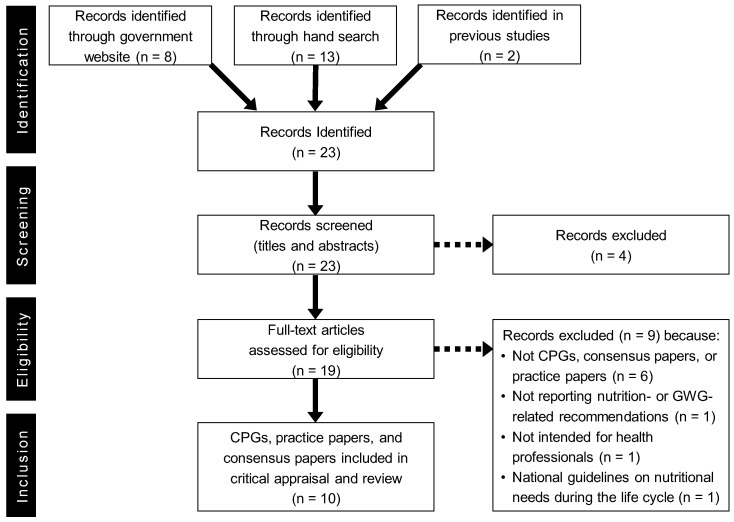
Flow diagram of the literature search process. CPG: clinical practice guideline.

Twenty-three potential documents were identified through the search process, of which four were excluded after screening titles and abstracts. Nineteen documents were assessed for eligibility using the criteria described in Table 1, of which nine were excluded for reasons shown in Figure 1. Finally, 10 CPGs were selected for the systematic review, four of which were obtained by searching national government websites, and the remaining six through arbitrary searching (Figure 1). As listed in Table 2, the selected CPGs were published in Sri Lanka [20], Myanmar [21], Australia [22], China [23], Japan [24], New Zealand [25,26], Philippines [27], Vietnam [28], and the Pacific Community (New Caledonia) [29]. Two CPGs were found in ‘New Zealand: Food and Nutrition Guidelines for Healthy Pregnant and Breastfeeding Women: A background paper (Food and Nutrition)’ [25] and ‘Guidance for Healthy Weight Gain in Pregnancy (Weight Gain)’ [26]. Two [20,21] of the 10 CPGs were from Southeast Asia, and the remaining eight CPGs [22,23,24,25,26,27,28,29] were from the Western Pacific region.

Data on the main characteristics of the 10 selected CPGs were extracted and are presented in Table 2. All CPGs, except for the Food and Nutrition CPG from New Zealand, have been published within the last decade. CPGs from Australia [22], Japan [24], and New Zealand [25] involved updating previously published guidelines. The CPG, published in New Caledonia [29] is the work of the Pacific Community, formerly the South Pacific Commission, the principal scientific and technical organisation of the Pacific Region. The Pacific Community is owned and governed by 26 Pacific Island countries and territories, of which all but Australia, France, New Zealand, and the United States represent small-island nations or territories in the South Pacific region. This CPG is intended for use in 22 other Pacific Island countries and territories, mainly composed of low- and middle-income countries. Most CPGs were written in English, with the exception of those from China [23], Japan [24], and Vietnam [27]. Almost half of the CPGs (*n* = 6) were developed by government bodies, whereas the others were developed by professional or scientific bodies. Half of the CPGs were developed with a focus on maternal care; however, all covered either GWG or diet/nutrition during pregnancy. Grading systems were applied to the recommendations in CPGs from Australia [22], Japan [24], and the Philippines [27].

Details of the AGREE II scores for each CPG are presented in Table 3. The scope and purpose, stakeholder involvement, and clarity of presentation domains scored more highly, while the rigour of development, applicability, and editorial independence domains scored less highly. Editorial independence was a particularly remarkable domain, with five CPGs assessed as 0%. The Australian CPG [22] was an exception, with the highest rating of more than 90% in all six domains and the highest rating in each domain among the 10 CPGs. As a result, there was considerable variability (from less than 10% to more than 90%) in the scores of the three domains listed above as lower-scoring domains. Finally, of the 10 CPGs, the CPG from Australia [22] was found to be the only guideline that was unanimously recommended for use without modification, with an overall quality rating of 100%. CPGs developed in Japan [24], and New Zealand [25,26] were recommended with modifications. Different sentiments were expressed about the CPG from Myanmar [21], and the remaining six CPGs were ‘not recommended’ for use. The CPGs from Sri Lanka [20], China [23], and the Philippines [27] received the lowest overall quality rating of 25%.

### 3.2. Overview of GWG Recommendations

Recommendations on total weight gain during pregnancy were found in the nine CPGs, as shown in Table 4. All nine CPGs provided weight gain (kg) by pre-pregnancy body mass index (BMI), which was divided into three or four categories. Six of these recommendations were clearly linked to the evidence base, all of which included GWG recommendations by the United States Institute of Medicine (IOM) in 2009 [30] or 1990 [31]. The remaining recommendations presented in the CPGs from Sri Lanka [20], Vietnam [28], and the Pacific Community [29] did not have links to the evidence base; however, the GWG values recommended in these CPGs [20,29] were almost identical to those in the 2009 IOM guideline. The CPG from Australia [22] included a category of pre-pregnancy BMI cut-off values for those with an Asian ethnic background, along with the recommendation by the IOM (2009). CPGs from Japan [24] and Vietnam [28] provided unique recommendations. The Japanese CPG included a GWG recommendation based on domestic evidence. It referred to the smallest lower limit of weight gain for women with a BMI less than 18.5 kg/m^2^ (9–12 kg) and a BMI between 18.5 and 25 kg/m^2^ (7–12 kg), and an individualised approach for women with a BMI greater than 25 kg/m^2^. The recommendation in the Vietnamese CPG was unique in that pre-pregnancy weight was used as the basis for calculating optimal weight gain, though its evidence base was unclear; for women with a pre-pregnancy BMI less than 18.5 kg/m^2^ or more than 25 kg/m^2^, the lower limit of increase was referred to as a percentage of pre-pregnancy weight, and for women with a normal BMI category (18.5–24.9 kg/m^2^), the GWG range was given in absolute terms in kg.

### 3.3. Overview of Nutritional Recommendations

Table 5 outlines the characteristics and spectra of the nutritional recommendations and issues covered by the CPGs. We focused on dietary advice, additional energy intake, and nutritional supplementation in the nine CPGs. Six CPGs included food-based advice, which covered foods to choose from and five covered foods to avoid or limit. Overall, recommendations on foods were scattered and covered a wide range of topics depending on the local food situation.

Some variation in recommendations for additional energy intake during pregnancy was observed; two guidelines did not mention additional energy intake, another two gave a single value for energy intake regardless of trimester, and the rest gave a value for each of the two or three trimesters. The guidelines referring to energy intake for each trimester provided recommendations as follows: 0–50 kcal/day for the first trimester, 250–340 kcal/day for the second trimester, and 300–450 kcal/day for the third trimester. The guidelines for a single value throughout pregnancy provided 300–360 kcal/day.

In terms of nutritional supplementation, folic acid was consistently recommended in all CPGs, and all nine CPGs recommended 400–800 μg of folic acid per day for all pregnant women; however, various statements were made as to when and for how long it should be taken. Additional folic acid was recommended in five CPGs for pregnant women with a history of pregnancy of a baby with neural tube defects. Iron supplementation was inconsistently recommended: seven CPGs recommended iron supplementation for all pregnant women, two recommended it under certain conditions, and one CPG did not mention iron supplements. Calcium supplementation was also inconsistently recommended; two CPGs were recommended for all pregnant women, three CPGs were recommended for those with specific conditions, such as pregnant women at risk of hypertension or women with low/no dairy intake, and the remaining five CPGs did not mention calcium supplementation. A minority of the CPGs also recommended iron and folic acid with vitamin C (Sri Lanka), vitamin B_1_ (Myanmar), and iodine (Australia) for all pregnant women. Vitamin B_12_ (Myanmar and New Zealand), omega-3 long-chain polyunsaturated fatty acids, and vitamin D (Australia) were recommended under certain conditions.

This study reviewed 10 CPGs relevant to 38 countries or territories from the Asia-Pacific region; 50% were published in high-income countries [22,24,25,26,29] In contrast, the previous review analysed 22 CPGs across the globe, 90% of which were from high-income countries [11]. One CPG included in this study was written by a regional scientific organisation for use in the 22 countries or territories, mainly composed of low- and middle-income countries of the Pacific region [29]. Although our study included guidelines from some countries that were included in previous studies [9,10,11], only one CPG from New Zealand (Food and Nutrition) overlapped because we selected the latest version of the CPG. We identified nine new CPGs on weight gain and nutrition during pregnancy.

We found that only one CPG developed in Australia was judged to be of high quality (≥70%). Most CPGs did not adhere to several components of AGREE II, reflected in low domain scores (a domain score of <50%), indicating limited quality. Three lower-scoring domains were identified: rigour of development, applicability, and editorial independence. The rigour of development was unsatisfactory because of the lack of systematic methods for searching and selecting evidence. The applicability of CPGs was rated poorly because of inadequate discussion about the application of facilitators and barriers and the calculation of implementation costs. Finally, the editorial independence domain had the lowest scores due to the lack of funding disclosure and competing interests involved in guideline development. These points must be considered when updating existing CPGs for better quality.

Nine of the 10 CPGs (90%) had GWG recommendations; the majority of the nine CPGs endorsed the GWG recommendation by the United States IOM in 2009 [30], while two unique recommendations were identified in the CPGs from Japan [24] and Vietnam [28]. It should also be mentioned that the CPG from Australia [22] included pre-pregnancy BMI cut-off values that matched with the Asian population. The recommendation of the IOM guideline (2009) was originally designed for a population consisting of white, Hispanic, and black individuals in the United States [30], and there were concerns about applying the IOM BMI cut-offs directly to the Asian population, as the body composition of Asians differs from that of other ethnic groups. Emerging evidence suggests that population-specific BMI cut-off points are more appropriate for optimal weight gain in some Asian populations [32,33,34,35]. Accumulating more evidence to determine optimal BMI cut-off points and weight gain specific to each ethnic group and incorporating this into future CPG recommendations is one of the key challenges in the Asia-Pacific region.

Regarding recommendations on diet and foods during pregnancy, six CPGs included foods to choose and five included foods to avoid or limit. Concrete advice was not extracted and synthesised in this study because it was found scattered at many different levels, with some CPGs describing recommendations very extensively. This may be due to the fact that the role of developing recommendations on food/diet varies from guideline to guideline. It seems that this role is often not assigned when the organisation level of the body is professional or scientific. As shown in Table 2, the guidelines for China, Japan, and the Philippines developed under the auspices of the Society of Obstetricians and Gynaecologists did not include advice on diet/food. In contrast, government-led CPGs have been more successful in including advice on food and diet with the involvement of nutrition experts and dietitians. To ensure that evidence-based dietary and food advice is provided to pregnant women in clinical practice, it would be worthwhile to consider involving individuals from all relevant professional groups in the guideline development group.

## 4. Discussion

With regard to nutritional recommendations, 7 out of the 10 CPGs (70%) had recommendations on additional energy intake, and 9 out of the 10 CPGs (90%) had some recommendations on nutritional supplementation, but these were not consistent across the Asia-Pacific region. In terms of the type of supplement recommended, folic acid was recommended by all nine CPGs for all women to prevent pregnancy with neural tube defects. However, there is some controversy regarding the other recommended supplements. Attitudes towards iron, in particular, varied from guideline to guideline; some recommended it for all pregnant women, some recommended it only in certain circumstances, such as in high risk of anaemia (Australia and New Zealand), and some did not mention it (Japan). Given the differences in nutritional status between countries, it is understandable that there is a wide variation in the recommendation; iron tends to be recommended for all pregnant women in developing countries, where food supplies are inadequate. Some other supplements, such as calcium, vitamins B_1_ and C, and iodine, have been recommended for all pregnant women in some CPGs. Others, such as vitamins B_12_ and D, multivitamin and mineral supplements, and omega-3 long-chain polyunsaturated fatty acids, have been recommended under certain conditions in some CPGs. Overall, there seems to be no consensus on the recommendations for nutritional supplementation other than folic acid for all pregnant women in the Asia-Pacific region. Therefore, the scope of reviews on nutritional supplementation should be extended in future CPGs.

The limitations of this study include the following: as this study was only able to search for guidelines in a limited number of languages (i.e., English, Chinese, Korean, Japanese, and Indonesian), it is possible that CPGs written in other languages may have been missed. Thus, it may have failed to provide a comprehensive picture of the entire Asia-Pacific region. Despite the linguistic limitations, this study was able to identify 10 CPGs, including CPGs written in English, Chinese, Japanese, and Vietnamese, out of 38 Asia-Pacific countries by searching grey literature. In the future, a search strategy covering more languages would enable certainty regarding full coverage of the Asia-Pacific region. Nevertheless, this study provides useful information on the quality of CPGs and variability of recommendations for GWG and/or maternal nutrition in the Asia-Pacific region, which was not covered well in previous studies [9,10,11].

Finally, although CPGs play an important role in clinical settings, healthcare professionals may also need to consider the cultural context of dietary intake. For instance, food taboos are typical cultural norms and are known in almost all human societies [36]. A recent review identified more than 50 foods that are taboo during pregnancy, including fresh meat, eggs, and various types of fruit and vegetables [37]. This appears to stem from ecological, medical, religious, and spiritual contexts [36]. Some of these could protect pregnant women from unhealthy diets, while others may cause deficiencies of certain nutrients. It is less clear how such cultural norms affect actual dietary intake during pregnancy vis-à-vis recommendations in CPGs. Such food taboos during pregnancy are often observed in Asian [38,39,40] and Pacific island [37] countries. How to consider these cultural norms in CPGs should be investigated in the contexts of the Asia-Pacific region in future research. Furthermore, this research did not consider the potential impact of the absence of guidelines on dietary intake and weight gain during pregnancy. CPGs can help to minimise disparities in clinical practice. Lack of specific CPGs may lead to variation in nutrition prescriptions during pregnancy. It may be valuable to examine why CPGs related to maternal nutrition and GWG are non-existent in some settings and examine the impact of this from the perspective of clinicians and women.

## 5. Conclusions

The present study found 10 CPGs on GWG and maternal nutrition in the Asia-Pacific region by searching the grey literature. According to the AGREE II appraisal, the quality of CPGs was generally low, with the exception of the CPG from Australia. There was wide variation in the recommendations on weight gain and nutrition during pregnancy. We identified several areas that could be improved in future guidelines. These findings provide an overview of the existing national and international recommendations on nutrition and weight gain during pregnancy in the Asia-Pacific region. This would provide useful information for wide authorities, including policymakers from health and welfare sections at local and national levels, health care professionals, and researchers.

## Figures and Tables

**Table 1:** PICAR statement for inclusion of CPGs.

Criterion	Description
(P) Population	Pregnant women
(I) Interventions	Any nutritional/dietary intervention for achieving a healthy pregnancy outcome
(C) Comparators	Any comparator or comparison. No key CPG content is of interest
(A) Attributes of eligible CPGs	(1) National and international CPGs, including consensus papers or practice papers (2) In a full-text format that is publicly available (3) Published since the year 2000 in any language (4) Issued from professional or governmental organisations in the Asia-Pacific region (5) Reporting nutrition- or/and GWG-related recommendations (6) Intended for health professionals (7) Latest version (8) With no restrictions on their quality, as assessed by the AGREE II instrument
(R) Recommendation characteristics and other considerations	Not applicable

PICAR: population and clinical areas, interventions, comparators, attributes of CPGs, and recommendation characteristics; CPG: clinical practice guideline; GWG: gestational weight gain; AGREE II: Assessment of Guidelines for Research Evaluation II.

**Table 2:** Characteristics of the CPGs for GWG and maternal nutrition.

Country/Region (Year of Publication)	CPG Title	Language	Name and Location of Publishing Organisation	Organisation Level of Body		Range of Topics Addressed			Intended Audience
				Governmental	Professional or Scientific	Maternal Care	GWG	Diet/Nutrition in Pregnancy	
Sri Lanka (2011) [20]	Maternal Care PackageA Guide to Field Healthcare Workers	English	Family Health Bureau, Ministry of Health, Colombo, Sri Lanka	✓		✓	✓	✓	Health workers who provide maternal and newborn care
Myanmar (2018) [21]	National Guidelines for Antenatal Care For Service Providers	English	Maternal and Reproductive Health Division, Ministry of Health and Sports, Nay Pyi Taw, Myanmar	✓		✓		✓	Service providers at all levels of the health system
Australia (2020) [22]	Clinical Practice Guidelines: Pregnancy Care 2020 Edition	English	Australian Government Department of Health, Canberra, Australia	✓		✓	✓	✓	All health professionals who contribute to pregnancy care, including midwives, obstetricians, general practitioners, Aboriginal and Torres Strait Islander health workers and allied health professionals
China (2018) [23]	Guidelines on preconception care and prenatal care (Translated)	Chinese	Obstetricians Group-Obstetrics and Gynecology Branch-Chinese Medical Association, Beijing, China		✓	✓	✓	✓	Clinicians
Japan (2020) [24]	Guideline for Gynecological Practice 2020 edition	Japanese	Japan Society of Obstetrics and Gynecology, Tokyo, JapanJapan Association of Obstetricians and Gynecologists, Tokyo, Japan		✓	✓	✓	✓	Physicians engaged in obstetric care
New Zealand (2006, revised 2008) [25]	Food and Nutrition Guidelines for Healthy Pregnant and Breastfeeding Women: A background paper (Food and Nutrition)	English	Ministry of Health, Wellington, New Zealand	✓			✓	✓	Health practitioners – including dietitians, nutritionists, midwives, doctors, nurses, primary health care providers, health promoters, and teachers
New Zealand (2014) [26]	Guidance for Healthy Weight Gain in Pregnancy (Weight Gain)	English	Ministry of Health, Wellington, New Zealand	✓			✓		Health practitioners
Philippines (2013) [27]	Clinical Practice Guidelines on Maternal Nutrition and Supplementation First Edition	English	Philippine Obstetrical and Gynecological Society, (Foundation), Inc. Metro Manila, Philippine		✓		✓	✓	The obstetrican-gynecologist, the general practitioner, the patient, the student, and the allied medical practitioner
Vietnam (2017) [28]	National Guidelines on Nutrition for Pregnant Women and Breastfeeding Mothers (Translated)	Vietnamese	Ministry of Health, Ha Noi, Vietnam	✓			✓	✓	Health professionals
The Pacific Community (New Caledonia) (2019) [29]	Pacific Guidelines for Healthy Eating During PregnancyA Handbook for Health Professionals and Educators	English	Public Health Division of the Pacific CommunityNoumea, New Caledonia	✓			✓	✓	Health professionals in the Pacific who provide advice related to family planning or pregnancies

CPG: clinical practice guideline; GWG: gestational weight gain.

**Table 3:** Critical appraisal of the CPGs on GWG and maternal nutrition using AGREE II.

Country/Region	AGREE II Domain (%)						Overall Quality	Recommendation (% of Reviewers)		
	Scope and Purpose	Stakeholder Involvement	Rigour of Development	Clarity of Presentation	Applicability	Editorial Independence		Yes	Yes, Needs Modification	No
Sri Lanka [20]	53	53	6	42	25	13	25			100
Myanmar [21]	69	53	17	53	40	13	42		50	50
Australia [22]	100	97	91	94	96	100	100	100		
China [23]	72	50	16	67	31	4	25			100
Japan [24]	81	81	61	86	13	92	67		100	
New Zealand (Food and Nutrition) [25]	81	61	24	75	44	0	58		100	
New Zealand (Weight Gain) [26]	97	53	25	72	40	0	50		100	
Philippines [27]	44	33	25	53	13	0	33			100
Vietnam [28]	28	28	4	61	10	0	25			100
The Pacific Community (New Caledonia) [29]	81	44	18	67	2	0	33			100

CPG: clinical practice guideline; GWG: gestational weight gain; AGREE II: Assessment of Guidelines for Research Evaluation II.

**Table 4:** Recommendations on GWG in CPGs.

Country/Region	Recommended as:	BMI (kg/m^2^)	Weight Gain (kg)	Evidence Based on
Sri Lanka [20]	Expected weight gain in kg	<18.5	12.5–18	Not mentioned
		18.5–24.9	11.5–16	
		25–29.9	7–11.5	
		≥30	≤6.8	
Australia [22]	IOM recommendations for weight gain in pregnancy	<18.5	12.5–18	NHMRC 2013 based on IOM 2009
		18.5–24.9	11.5–16	
		25–29.9	7–11.5	
		≥30	5–9	
	Recommendations for weight gain in pregnancy among women from Asian backgrounds	<18.5	12.5–18	NHMRC 2013 based on IOM 2009 and matched with Asian BMI cut-offs
		18.5–22.9	11.5–16	
		23–27.5	7–11.5	
		>27.5	≤7	
China [23]	Recommendations on the range of weight gain during pregnancy (Translated)	<18.5	12.5–18	American College of Obstetricians and Gynecologists. Committee Opinion No. 548 and No. 549
		18.5–24.9	11.5–16	
		25–29.9	7–11.5	
		≥30	5–9	
Japan [24]	Recommended values for weight gain during pregnancy (Translated)	<18.5	9–12	Japan Society for the Study of Obesity, Diagnostic criteria for obesity 2011; Ministry of Health, Labor and Welfare, Healthy Parents and Children 21
		18.5–25	7–12	
		>25	Individualised (standard: up to 5 kg)	
New Zealand (Food and Nutrition) [25]	Recommended total weight gain in pregnant women, by pre-pregnancy BMI (kg/m^2^)	<19.8	12.5–18	IOM 1990
		19.8–26	11.5–16	
		26–29	7–11	
		>29	6	
New Zealand (Weight Gain) [26]	Recommendations for total and average rate of weight gain during pregnancy, by pre-pregnancy BMI	<18.5	12.5–18	IOM and NRC 2009
		18.5–24.9	11.5–16	
		25–29.9	7–11.5	
		≥30	5–9	
Philippines [27]	Recommended total weight gain by pre-pregnancy BMI Classification of Pregnancy BMI	<18.5	12.7–18.1	IOM 2009
		18.5–24.9	11.3–15.9	
		25–29.9	6.8–11.3	
		≥30	5–9.1	
Vietnam [28]	Recommended weight gain	<18.5	At least 25% of pre-pregnancy weight	Not mentioned
		18.5–24.9	10–12	
		>25	At least 15% of pre-pregnancy weight	
The Pacific Community (New Caledonia) [29]	How much weight gain to recommend	<18.5	12.5–18	Not mentioned
		18.5–24.9	11.5–16	
		25–29.9	7–11.5	
		>30	5–9	

GWG: gestational weight gain; CPG: clinical practice guideline; BMI: body mass index; NHMRC: National Health and Medical Research Council; IOM: Institute of Medicine; NRC: National Research Council.

**Table 5:** Characteristics and spectrum of nutritional recommendations for pregnancy in the CPGs included in this review.

Country	Dietary Advice		Additional Energy Intake (kcal/day)	Nutritional Supplementation	
	Foods to Choose	Foods to Avoid/Limit		Supplement to Take for All Pregnant Women	Supplement to Take for Specific Conditions
Sri Lanka [20]	✓	Not mentioned	+360	Iron (60 mg) and folic acid (400 μg) with vitamin C (50 mg) per day after a period of amenorrhoea of 12 weeks for 6 months during pregnancy and 6 months after delivery Folic acid (5 mg) during first trimester Calcium (No mention of dosage)	Iron (double dose) for 3 months and monitor the progress for women with both moderate and severe anaemia Folic acid should be taken until the next pregnancy for women who have a history of having children with neural tube defects
Myanmar [21]	✓	✓	+300	Iron (60 mg) daily after the first trimester Folic acid (400 μg) daily starting in the first trimester and up to 37 completed weeks, and then twice daily up to delivery Vitamin B_1_ (10 mg) daily 1 month before pregnancy, during pregnancy, and 3 months after delivery	Vitamin B_12_ supplementation may be needed if a woman has a vegetarian or vegan diet Multivitamin and mineral supplements may be needed for women who are vegetarian, drink alcohol, use cigarettes or drugs, have been on a weight-loss program, and adolescents with poor nutrition Iron (double dose daily) for 3 months for women with moderate anaemia
Australia [22]	✓	✓	Not mentioned	Folic acid (400 μg/day) ideally from 1 month before conception and throughout the first 3 months Iodine (150 μg/day) Women with pre-existing thyroid conditions should seek advice from their medical practitioner before taking a supplement	Iron (80–300 mg weekly or 30–60 mg daily) supplementation to pregnant women based on their haemoglobin concentration at 28 weeks Calcium supplement for women at risk of hypertension (pre-eclampsia) Omega-3 long-chain polyunsaturated fatty acids (800 mg DHA and 100 mg EPA/day), if they are low in omega-3
China [23]	Not mentioned	Not mentioned	Not mentioned	Folate (400–800 μg/day) or folate-contained multivitamins from 3 months before pregnancy to 3 months of pregnancy Iron (60 mg/day) for women without anaemia Calcium (0.6–1.5 g/day)	Iron (100–200 mg/day) for women diagnosed with anaemia Folate (4 mg) every day for a woman who has previously given birth to a baby with neural tube defects
Japan [24]	Not mentioned	Not mentioned	+50 (1st tri) +250 (2nd tri) +450 (3rd tri)	Folic acid (400 μg) daily from before conception	Folic acid (4–5 mg/day) from preconception to the 11th week of pregnancy for women with a history of pregnancy with neural tube defects
New Zealand (Food and Nutrition) [25]	✓	✓	+0 (1st tri) +340 (2nd tri) +452 (3rd tri)	Folic acid (800 μg) daily for at least 4 weeks before and 12 weeks after conception	Calcium for women who consume little or no milk and milk products Iron if indicated by monitoring of iron status Vitamin B_12_ for pregnant and breastfeeding vegan women Folic acid (5 mg) for women at increased risk of having a pregnancy affected by an neural tube defect for at least 4 weeks before and 12 weeks after conception Vitamin D for covered women
Philippines [27]	Not mentioned	Not mentioned	+300 (2nd and 3rd tri)	Iron (30–60 mg) and folic acid (400 μg) daily throughout pregnancy	Iron is doubled (120 mg) if she is large, has twin foetuses, or begins supplementation late in pregnancy Folic acid (5 mg) prior to conception for women at high risk of having a child with neural tube defect
Vietnam [28]	✓	✓	+50 (1st tri) +250 (2nd tri) +450 (3rd tri)	Iron (60 mg) and folic acid (400 μg) every day through pregnancy until 1 month after childbirth, or multi-micronutrients, as required.	Not mentioned
The Pacific Community (New Caledonia) [29]	✓	✓	+300 (2nd tri) +400 or 450 (3rd tri)	Iron (30–60 mg) and folic acid (400 μg) * every day	Iron (120 mg) and folic acid (400 μg) * every day if diagnosed with anaemia Calcium (1.5–2 g) per day for women with low calcium intake

* Original sources show ‘400 mg’, but it is shown as ‘400 μg’ in this table, as ‘mg’ is apparently a mistake in the unit. CPG: clinical practice guideline. DHE: docosahexaenoic acid; EPA: eicosapentaenoic acid.

## Data Availability

The datasets used and analysed in the present study were taken from materials published online, which can be accessed from the URLs in the References section. A detailed protocol is available in PROSPERO, an international prospective register of systematic reviews. Available online: https://www.crd.york.ac.uk/prospero/display_record.php?RecordID=291395 (accessed on 31 January 2022).
